# Severe Hypokalemia Masquerading Myocardial Ischemia

**DOI:** 10.4021/cr222w

**Published:** 2012-09-20

**Authors:** Daniel Bogdanov Petrov, Svetlozar Ivanov Sardovski, Maria Hristova Milanova

**Affiliations:** a Department of Emergency Cardiology and Acute Internal Diseases “Pirogov” Emergency Hospital, 21 Totleben Ave, Sofia 1606, Bulgaria

**Keywords:** Hypokalemia, Myocardial ischemia, ST-segment depression

## Abstract

An advanced degree of body potassium deficit may produce striking changes in the electrocardiogram (ECG). These changes can result in incidental findings on the 12-lead ECG or precipitate potentially life-threatening dysrhythmias. Although usually readily recognized, at times these abnormalities may be confused with myocardial ischemia. The object was to report a case of severe hypokalemia mimicking myocardial ischemia. A 33-year-old, previously healthy man, presented to the Emergency Department (ED) with a progressive weakness and chest discomfort. The electrocardiogram showed a marked ST-segment depression in leads II, III, aVF, V_1_-V_6_. The initial diagnosis was non ST-elevation myocardial infarction. Echocardiography was normal and troponin levels were within normal limits. A more detailed history revealed that the patient had an episode of acute gastroenteritis with diarrhea and vomiting. Serum chemistries were notable for a potassium concentration of 1,8 mmol per liter. With aggressive electrolyte correction, the ECG abnormalities reverted as potassium levels normalized. Hypokalemia induced ST-segment depression may simulate myocardial ischemia. The differential diagnosis might be difficult, especially in the cases when ST changes are accompanied with chest discomfort.

## Introduction

Alternations in serum potassium levels can have dramatic effects on cardiac cell conduction and may lead to electrocardiographic changes. These changes can result in incidental findings on the 12-lead ECG or precipitate potentially life-threatening dysrhythmias. Although in some patients ECG abnormalities do not accompany serum potassium abnormalities the electrocardiogram is a useful screening tool for gauging the severity of the serum potassium abnormality and the urgency of the therapeutic intervention [1].

Electrocardiographic abnormalities secondary to potassium deficiency generally involve ST segment, T wave and U wave. Hypokalemia induced ST segment depression may simulate subendocardial injury or ischemia and ECG correlates of hypokalemia can be confused with myocardial ischemia. In many situations of clinical practice, differential diagnosis with myocardial ischemia might be difficult, especially in the cases when ST-T changes are accompanied with chest discomfort.

## Case Report

**Figure 1:**
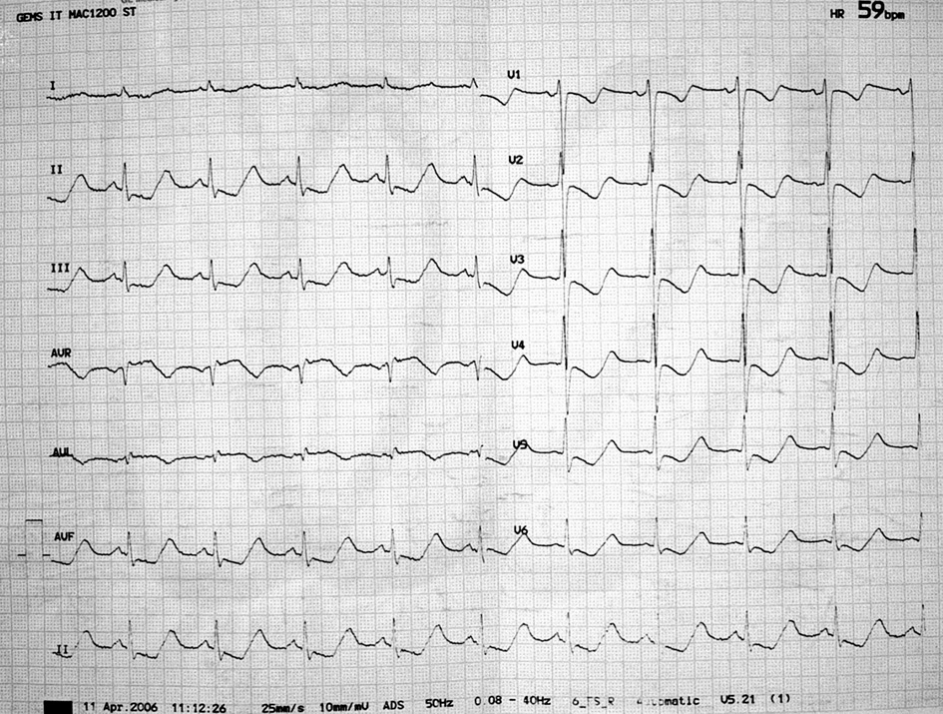
Admission electrocardiogram shows a marked ST-segment depression in leads II, III, aVF, V_1_-V_6._

**Figure 2:**
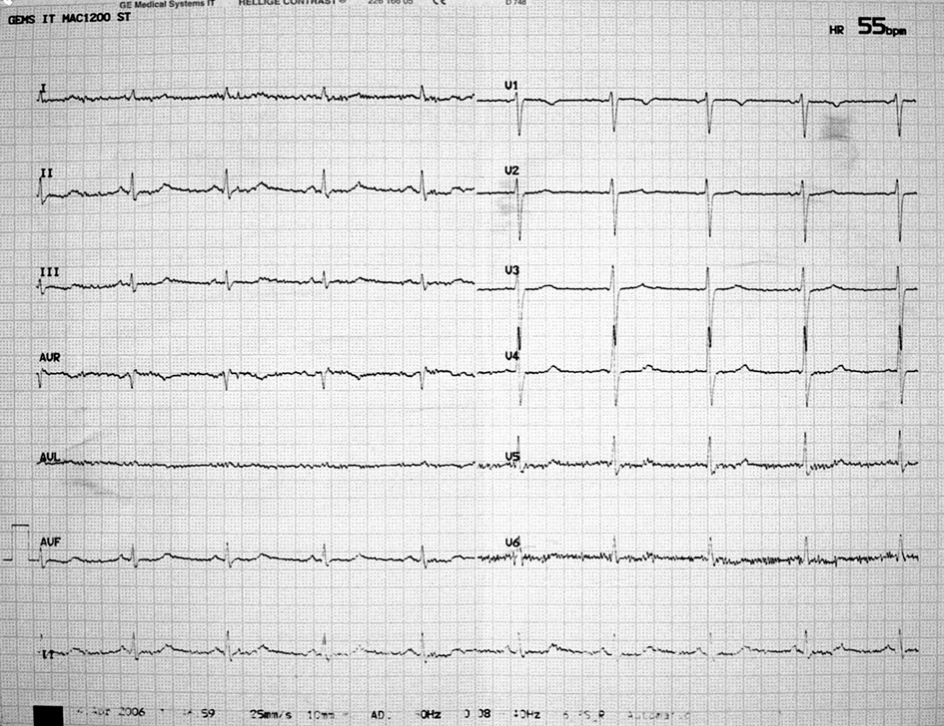
With aggressive electrolyte correction, the ECG changes revert to normal.

A 33-year-old, previously healthy man, presented to the Emergency Department with a two-day history of generalized, progressive weakness and chest discomfort. On the day of presentation the patient got up and tried to walk, but fell on his face due to his weakness. The 12-lead electrocardiogram (ECG) showed sinus rhythm at 59 per minute and a marked ST-segment depression in leads II, III, aVF, V_1_-V_6_ (Fig. 1). The initial diagnosis of the emergency physician was non ST elevation myocardial infarction, and the patient was admitted to our Cardiology Department for further evaluation. Transthoracic echocardiography was normal, with no evidence of regional wall motion abnormalities. A more detailed history revealed consumption of food of unknown origin during the holiday period, followed by a short episode of upper abdominal pain, diarrhea and repeated vomiting. Physical examination found a dehydrated patient with regular heart rate of 60 beats per minute and blood pressure of 80/50 mmHg. The pulmonary and cardiac examinations were unremarkable. On neurologic examination, he demonstrated intact sensation and motor strength, but had a slow gait. The abdomen was soft and non-tender, orienting diagnosis toward a case of acute gastroenteritis. Serum chemistries were notable for a potassium concentration of 1.8 mmol per liter and sodium concentration of 118 mmol per liter. Troponin levels were within normal limits. The patient was immediately started on potassium supplementation with continuous electrocardiographic monitoring and a central line intravenous infusion of 10 mEq of potassium chloride in 100 mL of dextrose 5% in water (D_5_W) per hour. His serum potassium levels were monitored every four hours. With aggressive electrolyte correction, the ECG changes reverted as potassium levels normalized (Fig. 2). At discharge, the patient’s serum electrolyte levels and electrocardiogram were normal.

## Discussion

Hypokalemia is a medical emergency and if it is not resolved quickly, decreased cardiac output and peripheral perfusion manifested in dysrhythmias and hypotension can lead to cardiac and respiratory arrest. Given the fact that untreated hypokalemia is associated with high morbidity and mortality, it is critical to recognize and treat that disorder promptly [2]. Early diagnosis and empiric treatment depend in many cases on the clinician’s ability to recognize the electrocardiographic manifestation of hypokalemia. Though laboratory tests are the gold standard tests for diagnosing changes in the serum electrolyte concentration, there may be delays in obtaining the results. The electrocardiogram may be a useful diagnostic tool if the clinician is aware of the possible changes resulting from abnormalities in the serum potassium concentration [3]. The electrocardiographic diagnosis of uncomplicated hypokalemia rests on ST-segment depression, a decrease in T-wave amplitude, prominent U waves and a U-wave to T-wave ratio greater than 1. The diagnosis is more difficult in a patient receiving digitalis, but the upsloping ST segment of digitalis ca be distinguished from that of hypokalemia [4]. The hypokalemic ECG index has been suggested as a predictor of hypokalemia. The index looks at the sum of the ST depression and the U wave in lead II and V_3_ to approximate serum potassium levels [5]. Electrocardiographic ST-segment depression require careful interpretation It is important to distinguish non-ischemic from ischemic ST depression, which sometimes may be an uneasy task in clinical practice. Non-ischemic causes of ST segment depression include right and left ventricular hypertrophy with a “strain” pattern in the right and left precordial leads, respectively. This is usually suggested by asymmetric ST depression. Digoxin therapy can also cause ST depression, sometimes resulting in a “reverse tick” type pattern. Conduction abnormalities can result in secondary ST segment changes (for example right bundle branch block, left bundle branch block and Wolf-Parkinson-White syndrome). Some cases of mitral valve prolapse and some diseases of the central nervous system can result in ST depression. Finally, hypokalemia and hypomagnesemia can also produce ST depression.

In our patient there was marked ST depression, bearing a close resemblance to the subendocardial injury or ischemia. But since the patient had normal levels of troponin and normal echocardiogram, the response to potassium supplement confirms that the ECG abnormalities were secondary to severe hypokalemia. The marked ST depression in the present case is a sign of advanced potassium depletion and it is important to note that ST-segment changes of this type are generally “functional” in type, without morphological substrate.

### Conclusion

Hypokalemia induced ST-segment depression may mimic myocardial ischemia. The differential diagnosis might be difficult, especially in cases when ST changes are accompanied with chest discomfort. The electrocardiographic abnormalities observed in the present case point out how closely a severe hypokalemia may simulate myocardial ischemia, if not actually lead to it.
